# Diabetes-Kidney-Heart Continuum and Its Implication on Therapeutic Management

**DOI:** 10.7759/cureus.88561

**Published:** 2025-07-22

**Authors:** Abdul Hamid Zargar, Jayagopal Pathiyil Balagopalan, Arpandev Bhattacharyya, Alan Almeida, Abhijit Taraphder, Sandeep Bansal, Sameer Dani, Nilakshi Deka, Sanjay Jain, Onkar C Swami

**Affiliations:** 1 Department of Endocrinology, Centre for Diabetes and Endocrine Care, Srinagar, IND; 2 Department of Cardiology, Lakshmi Hospital, Palakkad, IND; 3 Department of Diabetes and Endocrinology, Manipal Hospital, Bengaluru, IND; 4 Department of Nephrology, Parmanand Deepchand (PD) Hinduja Hospital and Medical Research Center, Mumbai, IND; 5 Department of Nephrology, Apollo Gleneagles Hospital, Kolkata, IND; 6 Department of Cardiology, Vardhman Mahavir Medical College and Safdarjung Hospital, Delhi, IND; 7 Department of Cardiology Services, Apollo Cardiovascular and Thoracic Institute, Ahmedabad, IND; 8 Department of Endocrinology, Apollo Hospital, Guwahati, IND; 9 Department of Medical Services, Alembic Pharmaceuticals Ltd., Mumbai, IND

**Keywords:** cardiovascular disease, chronic kidney disease, diabetes, dyslipidemia, guideline recommendations, pharmacotherapy

## Abstract

Type 2 diabetes (T2D), along with other co-morbidities (hypertension, hyperlipidemia, etc.), causes vascular complications and atherosclerosis, leading to heart or kidney damage. The timely detection of cardiovascular disease (CVD) and chronic kidney disease (CKD) risk helps in targeted treatment, thereby reducing hospitalization/death in people with T2D. The vascular complications of T2D, including the onset of CVD or CKD, have been widely studied. However, a clear understanding of the concurrent inter-relatability of diabetes-kidney-heart or diabetes-heart-kidney continuum would further assist the clinicians in preventing morbidity and mortality. The narrative review sought to outline the stages (“prevent,” “regress,” and “retard”), pathophysiological mechanism, and management of the continuum with defined patient profiles and associated risk factors. Pharmacotherapies with a focus on managing both cardiac and renal vascular changes (e.g., sodium-glucose transporter-2 inhibitors {SGLT-2is}, glucagon-like peptide-1 receptor agonists {GLP-1RA}, dipeptidyl peptidase-4 inhibitors {DPP-4is}, lipid-lowering therapy, and renin-angiotensin-aldosterone system {RAAS} blockers) have been discussed. In addition, new diagnostic approaches such as levels of B‐type natriuretic peptide (BNP), N‐terminal prohormone, cardiac troponin, cystatin C, and single-cell transcriptome sequencing, with proven accuracy for detecting vascular complications in the heart and kidney, have been summarized. The review underscores the importance of the early detection of the vascular complications in T2D with individual risk stratification for the initiation/continuation/switching of therapies, to enhance treatment adherence and outcomes.

## Introduction and background

Diabetes mellitus (DM) is a fast-growing major health problem, turning into a worldwide crisis with a global estimation of 537 million (in 2021, by the International Diabetes Federation). The number is further expected to rise to 783 million in 2045 [1]. Countries such as China, India, the USA, Pakistan, and Brazil have been the epicenters of diabetes in the last decade [2]. The overlapping co-morbid conditions of diabetes, hypertension, and dyslipidemia lead to atherosclerosis, endothelial dysfunction, structural remodeling, and vascular damage that cause cardiovascular diseases (CVDs) through microvascular and macrovascular complications [3]. The microvascular complications driven by hyperglycemia-induced oxidative stress, high vascular permeability, polyol accumulation, and tissue damage by advanced glycation end products (AGEs) develop diabetic kidney disease (DKD). The latter progresses to CVDs, with heart failure (HF) being the common outcome [3,4]. A monotonic increase in cardiovascular (CV) events, predominantly HF, is related to a decline in estimated glomerular filtration rate (eGFR) among people with type 2 diabetes (T2D) and chronic kidney disease (CKD) [5]. The change in eGFR in HF is attributable to longer vasoconstriction, low cardiac output, neurohumoral stimulation, and aggressive diuresis [6]. This impaired kidney or heart function in people with diabetes affects the function of the other, known as cardiorenal syndrome [4]. The concurrent existence of CVD, CKD, and T2D ultimately results in increased morbidity and mortality [7].

Glycemic control and the adequate management of elevated blood pressure (BP) and lipid levels have been suggested to increase life expectancy [7]. Given the heterogeneity in people with diabetes (hypertension or any other co-morbidities, CVD risk, metabolic syndrome, etc.), the management of cardiac and renal complications in such a population must be initiated by prioritizing their clinical profile [8,9]. There is an immediate need for focused research in key areas, including mechanisms linking metabolic syndrome with CVD and kidney dysfunction, associated risks, risk severity, and therapeutic decisions considering the risk factors [9]. Although the mechanism of T2D-initiated CVD or CKD onset has been widely studied, research on the vicious cycle consisting of these three entities that further affect or worsen the clinical outcomes is not well-established. Therefore, the rationale underpinning the study is to understand the link between diabetes, kidney, and heart functions. The narrative review sought to evaluate the pathophysiological mechanism of the diabetes-kidney-heart or diabetes-heart-kidney continuum and their global prevalence. Additionally, the efficacy and safety of different classes of pharmacotherapy in reference to clinical studies and guideline recommendations were summarized for the prevention and management of cardiorenal complications in people with coexisting diabetes, hypertension, and dyslipidemia.

Methodology

A comprehensive literature search was conducted for the inclusion of relevant studies (guideline/systematic review and meta-analysis/controlled trial/research article {prospective/retrospective/observational/cohort/post hoc analysis/cross-sectional study} and review article {consensus/comprehensive report/presidential advisory/narrative review}) on T2D, CVD, and CKD, published between 2014 and 2025. Briefly, the following combinations of keywords were used in PubMed and Google Scholar: cardiovascular disease, cardiorenal continuum, chronic kidney disease, diabetes, diagnostic tools, dyslipidemia, guideline recommendations, and pharmacotherapy.

The articles were independently screened by two authors, and the selection was later approved by all authors. Animal/molecular/in vitro studies, as well as protocol/conference proceeding/only abstract/short communication/editorial letter and articles not in the English language, were excluded. A total of 116 full-text articles were included, comprising 33 systematic reviews and meta-analyses, 35 research articles, 11 guidelines, 19 trials, and 18 review articles.

## Review

Diabetes triggers cardiorenal continuum

Epidemiology of Diabetes-Induced Cardiorenal Continuum

According to the Diabetes Atlas 2025, India ranks third in the number of adults with diabetes worldwide. Type 2 diabetes is predominant, with >90% global diabetes cases; 89.8 and 38.6 million Indians had diagnosed and undiagnosed (overwhelmingly T2D) diabetes, respectively, in 2024, with reported 0.33 million overall deaths [10]. Cardiovascular complications alone are responsible for 70% mortality in people with T2D [1]. The first CV event that occurs in T2D is HF, impacting the health of more than 30% of people [7]. Diabetes in older people (≥65 years) results in 68% death due to heart disease and 16% death by stroke [8]. Scientific evidence gathered from people with T2D across the world (2007-2017) showed 32.2% overall CV events: 29.1%, atherosclerosis; 21.2%, coronary heart disease; 14.9%, HF; 14.6%, angina; 10.0%, myocardial infarction (MI); and 7.6%, stroke. Coronary artery disease (CAD) and stroke were indicated as the cause of death in 9.9% of cases [11]. The reported prevalence of CVD and CKD among people with T2D in the USA was 21.6% and 24.1%, respectively; co-prevalence was 8.6% [12].

The chronic kidney disease that affects two out of every five people with T2D is still understudied in the diabetes setting. With CKD, the risk of major vascular events, stroke, and congenital heart disease (CHD) increases by 1.5-fold and death by twofold [13]. Stage 2 CKD (44.3%) was found to be the most common co-morbidity in people with diabetes (especially in older age groups) in the United Arab Emirates and Kuwait, followed by CVD (17.3%), CAD (15%), and HF (0.7%). A combination of CVD and CKD was present in 11.7% of people, while the co-prevalence of CAD and CKD was 9.7% [8]. A population-based survey of two major cities (Delhi and Chennai) in India showed 15.4% prevalence of CKD among people with DM; 47% had CKD with abnormal hemoglobin A1c (HbA1c) and hypertension. This reflected that one of every 12 people living in the largest cities of India has CKD [14]. Diabetic kidney disease is the leading cause of end-stage kidney disease (ESKD) and occurs in approximately 40% of diabetic people [5,7]. Individuals with diabetes are already at higher risk for developing CVD. The further development of DKD results in the onset of CVD in these people [15]. The risk of major adverse renal CV events was reported to be 97% in people with diabetes and HF, 93% in diabetes with CKD, 98% in diabetes with MI, and 89% in diabetes with stroke. Although 4/5 people with diabetes were free from CV and renal diseases, people having a history of these complications will eventually develop major adverse renal CV events; hence, preventive measures should be prioritized [16].

Renal Insufficiency in Diabetes Linked With CVD

Hyperglycemia in diabetes is the reason for the microvascular disease of the kidney and the eye, as well as the macrovascular disease of the heart and the brain [17]. People with diabetes and a history of CVD or HF are on the verge of developing albuminuria and ESKD, resulting in an increased risk of mortality [4]. Consistent CV events, particularly HF, decline eGFR and affect the kidney functions [5,7]. On the other hand, a progressive kidney disease may lead to HF and congestion [7]. A contemporary UK cohort witnessed the greatest risk of CVDs with eGFR of <30 mL/minute/1.73 m^2^ among people with diabetes and suggested that cardiorenal risk depends on the eGFR status-based CKD progression [5]. A study on 9,340 people with T2D and high risk of CVD reported composite renal outcome in 605, new-onset persistent macroalbuminuria in 376, and persistent doubling of serum creatinine in 184 people. Thirteen people died due to renal disease, and 120 underwent renal replacement therapy [18]. A randomized, placebo-controlled trial (Effect of Efpeglenatide on Cardiovascular Outcomes {AMPLITUDE-O} trial; n=4,076) reported that patients with ~15 years of diabetes and a history of CVD had a 21.8% incidence of current kidney disease (eGFR: 25 to <60 mL/minute/1.73 m^2^) [19]. The Empagliflozin Cardiovascular Outcome Event Trial in Type 2 Diabetes Mellitus Patients-Removing Excess Glucose (EMPA-REG OUTCOME) trial reported the incidence of CAD, peripheral artery disease, HF, and reduced eGFR (<60 mL/minute/1.73 m^2^) among 4,072/7,020, 603/7,020, 706/7,020, and 1,819/7,020 people with T2D, respectively [20]. In a longitudinal study by Wang et al., the risk of stroke with eGFR was assessed as 75-89 mL/minute/1.73 m^2^, 1.03 (95% CI: 0.90-1.18); 60-74 mL/minute/1.73 m^2^, 1.10 (95% CI: 0.94-1.28); 30-59 mL/minute/1.73 m^2^, 1.35 (95% CI: 1.15-1.59); and 15-29 mL/minute/1.73 m^2^, 1.64 (95% CI: 1.10-2.45), when adjusted for multiple factors. Reduced eGFR was the indicator of CHD and stroke in this study [15].

Pathophysiological Mechanisms of the Cardiorenal Continuum

**Figure 1:**
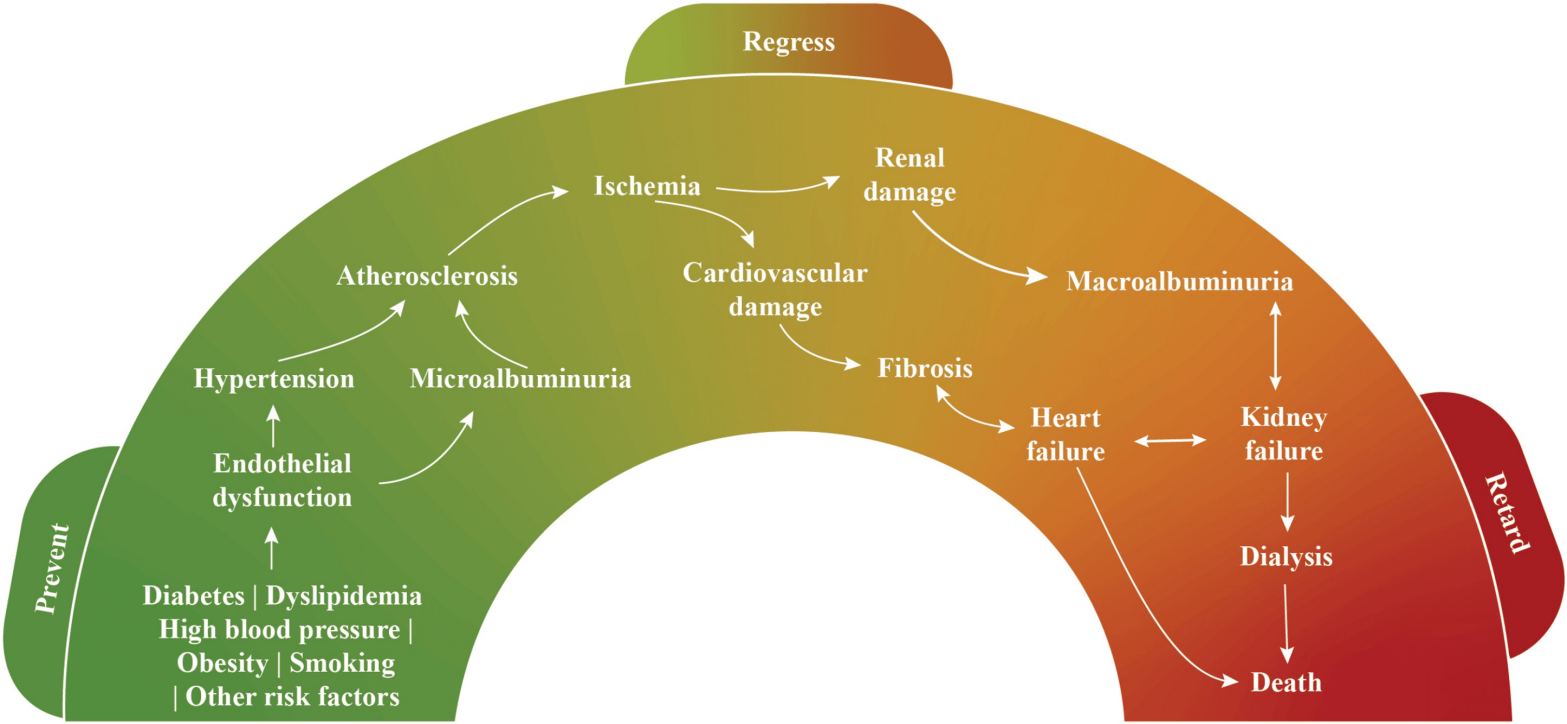
The cardiorenal continuum Credit: author’s own creation

The cumulative presence of old age, tobacco use, and hypertension or dyslipidemia confers to CVD emergence. Moreover, risk factors, such as elevated inflammatory markers and asymmetric dimethylarginine (ADMA), low nitric oxide bioavailability, and endothelial dysfunction, are related to atherosclerosis and contribute to renal impairment and CV events [15]. Accelerated calcification that occurs in people with CKD and/or diabetes is due to the imbalance of promoters (e.g. AGEs; alkaline phosphatase; apoptosis; bone morphogenetic proteins 2, 4, and 6; bone sialoprotein; calcium; hyperglycemia; inflammatory cytokines; matrix metalloproteinases; osteocalcin; oxidative stress; and phosphate) and inhibitors (e.g., bone morphogenetic protein 7, carbonic anhydrase, magnesium, matrix Gla protein, osteopontin, osteoprotegerin, and pyrophosphate) of calcification that predisposes them to HF and arrhythmias [21]. Coronary artery calcification leads to atherosclerotic CVD (ASCVD) [9]. Furthermore, in CKD, the elevation of inflammatory markers (e.g., C-reactive protein, D-dimer, fibrinogen, interleukin-6, and tumor necrosis factor α) and vascular/intercellular cell adhesion molecules, as well as the loss of renal mass-induced activation of the renin-angiotensin system (RAS), causes oxidative stress and atherosclerosis [15]. Cardiovascular risk factors result in endothelial dysfunction as indicated by increased BP or albuminuria [22]. Atherosclerotic vascular damage progresses with the advancement of the cardiorenal process, which in turn increases the overall risk for CVD and CKD. The determinants of renal dysfunction, anemia, secondary hyperparathyroidism, and atherogenic substance accumulation accelerate vascular disease at this stage. In case of the failed prevention of CV damage, CKD advances to overt nephropathy and CVD. Several vascular diseases may occur, including MI, angina, stroke, and renal failure, and subsequently lead to ESKD (Figure 1) [22,23].

The three clinically pertinent stages of the cardiorenal continuum are as follows [13]: (1) the clinical identification of the risk factors, in which the clinical approach is to “prevent” organ damage; (2) the clinical or laboratory identification of asymptomatic target-organ damage by albuminuria, decline in eGFR, left ventricular hypertrophy, etc., in which the clinical approach is to “regress” organ damage; and (3) symptomatic target-organ damage or established CVD/CKD, in which the clinical approach is “retard” organ damage.

The primary prevention of the CV risk factors, including glycemic control and the management of BP and dyslipidemia, can inhibit atherosclerosis. The secondary prevention or regression of albuminuria (a marker of renal and CV damage) should be targeted by means of suitable therapeutic approaches to delay cardiac and renal damage. Ultimately, palliative care should be provided in the last stage of the continuum [22].

New diagnostics

Asymptomatic left ventricular systolic and diastolic dysfunctions may develop even without established CVD incidence in people with T2D, which further aggravates the condition toward mortality [24]. Therefore, the early recognition of CVD in CKD could be helpful in therapy selection and in reducing CV events, hospitalization, and death. Several diagnostic approaches are available, including electrocardiogram, echocardiography, myocardial scintigraphy, nuclear magnetic resonance, computed tomography, and ambulatory heart rhythm monitoring, for the timely diagnosis and treatment of CVD in high-risk populations [25].

Natriuretic peptides or cardiac troponin are used for the detection of stage B HF (i.e., without symptoms but structural/functional cardiac abnormalities) in people with diabetes for the early implementation of effective therapeutic strategies; serial measurements of both detect people at high risk for incident HF [26]. B‐type natriuretic peptide (BNP) and N‐terminal prohormone BNP (NT‐proBNP) levels may predict the adverse prolonged outcome in individuals who have a history of both known and unknown incidence of HF [27]. High-sensitivity cardiac troponin T of ≥14 ng/L and NT-proBNP of ≥125 pg/mL indicate subclinical HF in people with CV-kidney-metabolic syndrome [9]. Therefore, a high-risk population with CKD, diabetes, or microalbuminuria is more likely to benefit from natriuretic peptide or high-sensitivity cardiac troponin testing [26]. Acute MI is prevalent among patients with CKD, who could have atypical symptoms and no ST-segment elevation. High diagnostic accuracy in such patients can be obtained with cardiac troponin measurements. Other non-nephrotoxic imaging techniques such as echocardiography, single-photon emission computed tomography, or coronary angiography (after reno-protective treatment) could be used in such kind of patients [28]. However, elevated troponin should not be used as a screening tool for HF as it predicts MI through any mechanism [29]. Deep learning algorithms, annotating two-dimensional videos along with Doppler modalities, can quantify the appropriate functioning of the different cardiac parameters. The automated workflow that generated interpretable annotations when integrated in echocardiograms can detect HF accurately in different cohort ethnicities, similar to the manual measurements [30]. Ghorbani et al. reported that the use of convolutional neural networks on a large Stanford echocardiography database and deep learning applied to echocardiography helped in identifying enlarged left atrium, left ventricular hypertrophy, ejection fraction (EF), left ventricular end systolic and diastolic volumes, and the presence of pacemaker leads and in predicting systemic phenotypes of age, sex, weight, and height [31]. Moreover, mid-regional pro-adrenomedullin biomarker is used for determining the therapeutic effectiveness of any antihypertensive drug in people with CKD and uncontrolled BP [32].

The creatinine equation is generally used in determining eGFR and urinary albumin-to-creatinine ratio (UACR) to predict kidney damage. The addition of cystatin C provides greater accuracy, particularly in people with altered creatinine production/metabolism [33]. In patients with DM, serum cystatin C (cutoff value of 82, sensitivity of 81.4, and specificity of 82.4) can act as a better early marker of DKD, compared to albumin excretion ratio and serum creatinine [34]. Besides the current diagnostic methods for kidney disease, the emergence of liquid biopsy, single-cell transcriptome sequencing, and miRNA analysis helps in detecting early-stage kidney diseases and monitoring the treatment responses. Liquid biopsy examines abnormal kidney function markers, the exosomes, and miRNAs to detect DKD in a less invasive approach than the traditional biopsies. However, their use in routine practice should be validated with clinical research [35].

Therefore, the accurate diagnosis of metabolic risk factors and renal and cardiac dysfunctions is crucial for opting for interventions and preventing the disease progression [9].

A holistic approach for preventing the diabetes-kidney-heart continuum

The management of CV-kidney-metabolic health through selecting risk prediction algorithms and holistic approaches among the growing array of healthcare therapies, especially in the three stages of the continuum, needs proper guidance.

Optimal Glycemic Control

Improved glycemic control reduces the occurrence of diabetic nephropathy [18]. The conventional DKD management strategies include lifestyle modification along with BP control with the four pillars, the RAS blockers, sodium-glucose transporter-2 inhibitors (SGLT-2is), glucagon-like peptide-1 receptor agonists (GLP-1RA), and non-steroidal mineralocorticoid receptor antagonists (MRAs) [36]. Landmark studies on diabetes and CVDs have supported therapeutic management with SGLT-2is, GLP-1RAs, dipeptidyl peptidase-4 inhibitors (DPP-4is), and other antidiabetic drugs, as discussed below.

Sodium-Glucose Transporter-2 Inhibitors

The Kidney Disease: Improving Global Outcomes (KDIGO) guidelines recommended SGLT-2i (and metformin) to be used as the first-line treatment for preventing CV events in patients with T2D and CKD, if eGFR (≥20 mL/minute/1.73 m^2^) allows [37,38]. It provides reno-protection by reducing the decline in eGFR and preventing the new onset and regression of macroalbuminuria [13]. A network meta-analysis on patients with T2D found a remarkable effect of dapagliflozin and canagliflozin on renal and CV outcomes and in reducing all-cause mortality, independent of gender [17]. A meta-analysis recorded a notable reduction in risk for CV death with empagliflozin, low risk for major adverse CV events (MACE) and the emergence or progression of kidney disease with canagliflozin, and reduced risk for hospitalization for HF (hHF) with dapagliflozin in patients with T2D [39].

The SCORED trial (n=19,188) revealed that sotagliflozin lowered the risk of CV events in subjects with T2D, CKD (median eGFR: 44.5 mL/minute/1.73 m^2^) and additional CV risks [40]. Empagliflozin, given together with standard care in people (n=4,687) with T2D and established CVD, decreased the rate of CV death and nonfatal MI or stroke [20]. However, the effects of SGLT-2is vary by patient profile as maximum benefits in reducing HF were noted in people with lower eGFR, whereas moderate benefits on MACE were reported in people with established ASCVD [41].

Dapagliflozin reduced kidney failure, CV death, or hHF in individuals with or without T2D (DAPA-CKD trial; n=4,304), independent of HF history [42]. The DAPA-HF trial (n=4,744) on patients with HF (class II/III/IV and EF of ≤40%) and with or without T2D reported the reduced risk of worsening HF or death from CV causes with dapagliflozin treatment, provided in addition to the recommended therapy [43]. Similarly, patients with HF (class II/III/IV and EF of ≤40%, with or without DM) of the EMPEROR-Reduced trial (n=3,730) showed a lower risk of CV death or hHF with empagliflozin, given together with recommended HF treatment [44]. Patients with diabetes and recent worsening HF of the SOLOIST-WHF trial (n=1,222) demonstrated low CV deaths and hHF with sotagliflozin therapy when initiated pre- or post-discharge [45]. Sodium-glucose transporter-2 inhibitors are generally well-tolerated but often result in adverse events that are mostly manageable and do not require treatment discontinuation. A systematic review reported diarrhea with the use of sotagliflozin; diabetic ketoacidosis with sotagliflozin and canagliflozin; acute kidney injury (AKI) and acute kidney failure with empagliflozin; and genital infections, volume depletion, and lower limb amputations with empagliflozin and canagliflozin [1].

Glucagon-Like Peptide-1 Receptor Agonists

In case the HbA1c goal is not achieved with the first-line therapy, then GLP-1RA (dulaglutide) can be considered in obese (type 2) people with diabetes but without congestive HF. However, the co-administration of GLP-1RA with insulin or sulfonylurea is not advisable to avoid the incidence of hypoglycemia [46]. A network meta-analysis of 12 trials reported a lower risk of kidney-specific composite outcome and worsening albuminuria with dual glucose-dependent insulinotropic polypeptide/GLP-1RA (tirzepatide) in patients with T2D [47]. Glucagon-like peptide-1 receptor agonists result in decreased CV death, stroke, or MI [48], along with reduced all-cause mortality, hHF, and risk of kidney dysfunction in people with T2D [48,49]. A post hoc analysis on T2D patients, who were at high risk of CVD, has shown a greater risk of MACE with kidney impairment than without. A reduction in HbA1c with semaglutide regardless of baseline eGFR (<45 and ≥45 to <60 mL/minute/1.73 m^2^) and UACR (≥30 mg/g) indicated its CV benefits [50].

The Evaluation of Lixisenatide in Acute Coronary Syndrome (ELIXA) trial of lixisenatide (given in addition to the usual care, n=3,031) in people with T2D, macroalbuminuria, and recent coronary event found a decrease in UACR [51]. Efpeglenatide, another GLP-1RA, reduces the risk of CV events among people with T2D, a history of CVD or current kidney disease, and one other CV risk factor [19]. Sodium-glucose transporter-2 inhibitors and GLP-1RA can be used in case of CVD or renal complications [52]. Using exenatide and SGLT-2i in parallel or in relatively close sequence has shown benefits on mortality, as well as CV and renal outcomes in patients with T2D and a history of CVD and HF (EXSCEL trial, n=14,752) [53].

In the LEADER trial (n=4,668), liraglutide (added to standard care) showed a notable reduction in the primary composite outcome, including CV death, nonfatal MI, or stroke among patients with T2D and established CVD and/or CKD stage 3 or higher [54]. The reduced risk of DKD progression and the low rate of the new onset of macroalbuminuria were reported with liraglutide treatment in T2D patients with eGFR of 15 to <30 mL/minute/1.73 m^2^ and a high risk of CVD (n=4,668), who were already receiving the usual care. Increase in UACR was less (17%) with the treatment [18]. Similarly, in people with T2D and established CVD and/or CKD stage of ≥3 (SUSTAIN-6 trial, n=1,648), semaglutide (on top of standard care) was reported to lower the rate of CV death and nonfatal MI or stroke [55]. A systematic review and meta-analysis on GLP-1RAs demonstrated improvements in glycemic control, weight reduction, and CV outcomes among individuals with T2D and advanced CKD and ESKD; however, the rate of gastrointestinal side effects such as nausea and vomiting was higher [56].

Dipeptidyl Peptidase-4 Inhibitors

Dipeptidyl peptidase-4 inhibitors, linagliptin and saxagliptin, provide reno-protection apart from glycemic control [13]. A systematic review and meta-analysis of 17 randomized controlled trials supported the use of DPP-4is (sitagliptin/vildagliptin/alogliptin/linagliptin) in patients with T2D and the presence or absence of albuminuria, as they potentially reduce UACR and decline in eGFR [57]. Another systematic review and meta-analysis (of eight randomized controlled trials) found no improvement in eGFR or mortality in patients with T2D but retard albuminuria progression with 52 weeks of DPP-4i therapy [58].

Sitagliptin added to the usual care showed a lower risk of MACE, hHF, or death in people (n=7,332) with T2D and established CVD of the TECOS study [59]. Moreover, data from the Taiwan National Health Insurance Research Database (n=3,750) showed that the risk of CV death, nonfatal MI or stroke, and hHF was not increased with vildagliptin in patients with T2D (n=1,250) having recent acute ischemic stroke or coronary syndrome [60]. In patients (n=7,348) with DM and AKI (eGFR: 70.5±35.5 mL/minute/1.73 m^2^), DPP-4i treatment resulted in the decreased risk of mortality, MACEs, major adverse kidney events (MAKEs), and re-dialysis [61].

The CARMELINA trial on people with T2D and nephrotic-range proteinuria (baseline UACR of >2,200 mg/g creatinine with any eGFR) showed a reduction in albuminuria and HbA1c with linagliptin and standard of care treatment; the therapy did not affect the CV or kidney risks [62]. Sitagliptin, when administered to 205 patients with T2D, CKD, or ESKD (on hemodialysis) and hospitalized for acute MI, resulted in no increase in CV death, ischemic stroke, or hHF risks [63].

Other Antidiabetic Drugs

In people with T2D, risks of hypoglycemia and weight gain with insulin and sulfonylurea rendered metformin the drug of choice in reducing the macrovascular risk of MI and CV death [52]. A cohort study on people with T2D and high CV risk (n=12,156) showed lower rates of all-cause mortality with metformin but not the composite endpoint of CV death, MI, or stroke. The findings were most evident in people without prior HF or moderate-to-severe CKD (eGFR: ≤45 mL/minute/1.73 m^2^) [64]. A retrospective cohort study reported reduced risk of diabetic nephropathy, MACE, and MAKEs with the use of metformin [65]. The EDGE study in patients (n=66) with T2D reported a similar effect of ɑ-glucosidase inhibitor (voglibose) and sitagliptin in improving endothelial dysfunction. The study reported diarrhea, edema, nausea, and abdominal fullness after 12 weeks of voglibose treatment [66]. The thiazolidinedione drug class (particularly pioglitazone) is responsible for lowering HbA1c and CV events and for improving insulin sensitivity in T2D [52]. As insulin clearance is carried out by the kidneys, reduced eGFR in CKD may result in hypoglycemia; hence, the modifications of insulin should be contemplated to reach glycemic targets [46]. Additionally, insulin therapy is believed to be associated with increased risk of ASCVD and hypoglycemia [52]. The reason could be the insulin resistance in people with T2D, receiving insulin therapy. A retrospective real-world study on hospitalized patients with T2D (n=2,356) reported insulin therapy linked to a high risk of carotid atherosclerotic plaque, including macrovascular complications, even after adjusting for the CV risk factors [67].

The low risk of all-cause mortality and CV events was found with metformin in people with T2D and mild or moderate CKD (eGFR: ≥30 mL/minute/1.73 m^2^) [68]. However, in mild-to-moderate CKD, the use of metformin should be expanded with caution depending on the eGFR and regular follow-up of kidney function [69]. Additionally, thiazolidinedione may improve glycemic control similar to other antidiabetic medications in patients with diabetes and renal dysfunction [70].

The Food and Drug Administration (FDA) has approved the use of metformin in individuals with HF (in 2006), as well as with kidney disease (in 2016) [52]. The retrospective cohort study by Yi et al. reported lower risks of MACEs and MAKEs in CKD patients (stages 3A, 3B, and 4) with the continuous use of metformin than no or discontinued use [65]. However, prolonged metformin use-associated adverse effects are diarrhea, bloating, abdominal cramping, and vitamin B12 deficiency [46]. Sulfonylureas and DPP-4is are the most commonly used glucose-lowering treatments in patients with T2D and advanced CKD (stages 3b-5). A real-world study on this population reported similar safety profiles of DPP-4i (n=1,204) and sulfonylureas (n=1,204) regarding renal and CV outcomes and all-cause mortality. However, different DPP-4is are preferred over sulfonylureas due to a lower risk of hospitalized hypoglycemia and all-cause mortality [71]. Thiazolidinediones can be used in CKD as they are not cleared by the kidney, but fluid retention is a major adverse effect; therefore, the drug should not be used in advanced HF [46].

Lipid-Lowering Therapy

The management of low-density lipoprotein-cholesterol (LDL-C) is the primary therapeutic goal in people with ASCVD. Besides LDL-C, the management of non-high-density lipoprotein-cholesterol (HDL-C) is particularly recommended in the Indian population by the Lipid Association of India. The recommended targets for LDL-C and non-HDL-C are <50 mg/dL and <70 mg/dL, respectively, in people with T2D and CVD or ≥2 additional major CV risk factors [13].

Statin and Non-statin Agents

In case of very high triglyceride levels (>500 mg/dL), fibrates and omega-3 fatty acids could be considered, followed by statin therapy to reach the LDL-C and non-HDL-C targets [13]. In addition, bile acid sequestrants are used for lowering cholesterol and improving glycemic control in people with impaired liver and kidney function due to their low systemic toxicity rate [72]. Cholesterol-lowering therapy with bempedoic acid helps in reducing CV risk in people with diabetes (with/without CVD), as evidenced by the Cholesterol Lowering via Bempedoic Acid (CLEAR) trial (n=13,970) [73]. Advanced lipid-lowering therapies such as proprotein convertase subtilisin-kexin type 9 (PCKS9) inhibitors, particularly in combination with statins, have higher LDL-C target achievement rates in T2D patients [74].

Fibrates are beneficial in people with moderate CKD and co-morbidities (DM, hypertension, atrial fibrillation, peripheral artery disease, HF, MI, and stroke), who had never received statin or other lipid-lowering agents to reduce CV events by decreasing triglycerides and increasing HDL-C levels [75]. A meta-analysis of 21 randomized studies observed that fibrates alone or with statin reduced albuminuria progression and increased albuminuria regression in people with/without diabetes and mild CKD [76]. In CKD patients, PCKS9 inhibitors reduce LDL-C and CV events, when added to existing statin therapy; however, their safety and efficacy in severe CKD and ESKD are not established [77]. A meta-analysis of 19 randomized trials reported reduced CV morbidity in secondary-prevention patients having a history of ASCVD (CAD, peripheral artery disease, or cerebrovascular disease) with intensified statin therapy alone or added to PCSK9 inhibitors or ezetimibe [78]. Statin intolerance and ezetimibe side effects led to the use of PCSK9 inhibitors for the secondary prevention of patients with CV risk factors and established CVD, who achieved the recommended LDL-C targets with the therapy [79]. Moreover, a meta-analysis of five randomized trials emphasized non-statin lipid-lowering therapy with a small-interfering RNA, inclisiran, that reduces LDL-C, PCSK9, and total cholesterol levels in individuals with ASCVD [80]. Inclisiran is approved by the FDA and the European Medicines Agency. This cholesterol-lowering therapy was reported to alter total cholesterol, HDL-C, non-HDL-C, apolipoprotein B, and lipoprotein(a) and decrease pre-specified CV endpoints (cardiac death, signs or symptoms of cardiac arrest, nonfatal MI, or stroke) at 18 months [81].

A nested case-control study on 15,830 patients with CKD recorded a reduced risk of MACE with current fibrate use (especially pemafibrate) and suggested its effective use in CKD patients with eGFR of 15-30 mL/minute/1.73 m^2^ [82]. A meta-analysis of 14 trials on dialysis patients reported a reduction in triglyceride and LDL-C levels with omega-3 supplementation but no change in total cholesterol, HDL-C, and albumin levels [83]. In addition, bile acid sequestrants can be used in T2D and ESKD together with metformin and statin due to their non-absorbable nature; however, associated gastrointestinal adverse events, especially constipation, should be considered [72]. The FOURIER trial (n=27,564) on individuals with atherosclerosis and advanced CKD demonstrated greater reduction in the composite of CV death, MI, and stroke with evolocumab (PCSK9 inhibitor) treatment [84].

Nicotinic Acid

Lipid-lowering agent niacin alone or in combination with statin significantly improved the lipid profile of patients with T2D, but long-term use increased fasting plasma glucose. Hence, regular glucose monitoring is required during long-term treatment [85]. A meta-analysis of 11 randomized trials concluded that niacin provides CV benefits when added to ongoing statin therapy; however, it is associated with a 35% risk of developing diabetes [86]. The 2025 American Diabetes Association (ADA) “Standards of Care in Diabetes” recommended not to use niacin-statin combination therapy due to the high risk of stroke, with additional side effects and additional CV benefits compared to statin therapy [87].

Blood Pressure and Albuminuria Control: Renin-Angiotensin-Aldosterone System (RAAS) Blockers

The renin-angiotensin-aldosterone system (RAAS) plays a crucial role in BP control and renal functioning, particularly in slowing DKD progression and lowering proteinuria and the risk of overt nephropathy. The reno-protective effects of the RAAS blockers support their use in hypertension and CKD, especially when people have proteinuria [88].

Angiotensin-Converting Enzyme Inhibitors (ACEis) and Angiotensin Receptor Blockers (ARBs)

Angiotensin-converting enzyme inhibitors (ACEis) reduce all-cause and CV mortalities, as well as MACE in people with DM; on the contrary, angiotensin receptor blocker (ARB) has no such benefits on CV outcomes [89]. A cohort study on people with diabetes found comparable effects of ACEi (n=34,043) and ARB (n=23,772) on hospitalization for AKI or hyperkalemia, long-term dialysis, and all-cause, CV, and non-CV deaths [90]. Another cohort study found similar effects of ACEi (n=55) and ARB (n=68) on UACR in people with hypertension and T2D; slightly increased eGFR was recorded with ARB, compared to ACEi [91]. The antiarrhythmic effects of ARBs are obtained through alterations of ion channel activity, refractoriness, and sympathetic tone and the inhibition of angiotensin II activity in the left atrium. This in turn affects the atrial stretch, inflammation, interstitial fibrosis, and structural remodeling and consequently the persistence and recurrence of atrial fibrillation. Telmisartan has a promising role in preventing paroxysmal to persistent atrial fibrillation [92]. Therefore, in people with diabetes and hypertension, choices of ARBs are telmisartan and losartan for reducing CV risk factors [93].

Among people with CVD or CKD, ACEi provides additional reno-protective effects against long-term dialysis, compared to ARBs [90]. Similarly, in CKD patients of a 15-year cohort study, a lower incidence of ESKD (1.39% versus 2.34%, p=0.008) was noticed with ACEi (n=6,898) treatment than ARB (n=12,758); the result was similar (0.30% versus 0.37%, p=0.11) in those without CKD. However, the ACEi group reported a higher risk of stroke than the ARB group [94]. In patients with DM and albuminuria, both ACEis and ARBs reduced the doubling of serum creatinine; however, ARBs were preferred for reducing the risk of ESKD by 23% [95]. Monotherapy or combination therapy of ARB demonstrated optimum BP control and improved proteinuria in people with hypertension and CKD, with or without diabetes [88]. Telmisartan is the preferred ARB in people with a history of atrial fibrillation, while losartan is recommended in people at a higher risk of stroke [93].

In patients with peritoneal dialysis, ACEis and ARBs reduce the loss of residual renal function, though in dialysis patients, the RAS blockers do not reduce CV events [96]. A cohort study (nationwide Korean Heart Failure registry) on moderate-to-severe CKD patients, hospitalized for acute HF (n=1,601), reported a reduction in one-year mortality and HF readmission with the initiation of ACEi or ARB treatment at discharge. The study also suggested considering RAS blockers in people with above-moderate renal insufficiency but not with ESKD [97].

Mineralocorticoid Receptor Antagonists

Mineralocorticoid receptor antagonists improve arterial stiffness and endothelial function by exerting beneficial effects on pulse wave velocity, augmentation index, and flow-mediated dilation [98]. In patients with hypertension and DM or overt albuminuria or proteinuria, the combination therapy of MRA with RAS blocker reduces BP and UACR but results in slightly elevated potassium concentrations [99].

Non-steroidal MRAs reduce BP, the risk of composite kidney, and CV outcomes and help in the remission of proteinuria and hence can be used for cardiorenal protection in people with CKD [100]. Finerenone, an MRA, reduces the risk of MACE, the progression of kidney disease, and hHF in people with established T2D and ASCVD [101]. Other studies reported similar findings of finerenone in people with T2D and CKD [102,103].

The American College of Cardiology/American Heart Association/Heart Failure Society of America (ACC/AHA/HFSA) clinical guideline (2022) recommended MRA (spironolactone or eplerenone) in HF with reduced EF (classes II-IV), eGFR of >30 mL/minute/1.73 m^2^, and serum potassium of <5.0 mEq/L to reduce morbidity and mortality [104]. The 2023 European Society of Cardiology (ESC) guideline also recommended MRAs for patients having HF with reduced EF and diabetes to reduce the risk of hHF and death [105]. The FINEARTS-HF trial on 6,001 patients with HF (left ventricular EF of ≥40%) reported a reduction in CV death and the worsening of HF events with finerenone treatment concomitantly used with SGLT-2i [106]. Among older patients with HF and DM or acute renal insufficiency, MRA reduced the risk of hHF but was associated with a high risk of hyperkalemia and acute renal insufficiency, particularly in those with preserved EF. This indicated the limited safety of MRAs in the older population [107]. Therefore, routine potassium monitoring and dose reduction or the temporary discontinuation of finerenone can be opted for protecting the renal and CV systems from hyperkalemia [108].

Strategies to Improve Treatment Adherence

Despite the availability of evidence-based recommendations, factors such as high prescription costs and insufficient guidance from healthcare professionals may negatively impact adherence to a treatment, as well as the health of the community. Therefore, reduction in healthcare cost and easy accessibility to healthcare providers need to garner the same attention as research, in the real-world setting [109]. The FDA focused on ways to improve the appropriate use of the approved medications that include access to generic medications, the active monitoring of regulated products, health literacy, medication guides, and public workshops for providing up-to-date product information [110]. Another approach can be considering single-tablet combination therapies for improving patient adherence and clinical outcomes while providing economic advantages [111]. Moreover, patient-centered education and patient-reported experience or perception may aid in developing new routines to improve treatment adherence [112].

Guideline recommendations for preventing and managing cardiorenal complications in patients with diabetes

In patients with T2D and established CVD or high CV risk, the 2022 ACC/AHA/HFSA guideline recommended treatment with SGLT-2i for the prevention of hHF [104]. The ADA clinical practice recommendations (2025) suggested GLP-1RA and SGLT-2i combination therapy in people with T2D and those with the risk of or established ASCVD to reduce adverse CV risk and renal events [87]. On the contrary, the 2023 ESC guidelines expressed their reservation on the use of GLP-1RA and SGLT-2i combination therapy for obtaining favorable cardiorenal outcomes in patients with T2D [105]. An Indian practice standard and management algorithm indicated ACEis for people with diabetes and stable angina, hypertension, HF, or early CKD. In addition to ACEi, high-intensity statin therapy was also recommended in people with diabetes and stable CAD. Oral antidiabetics such as metformin, gliclazide, gliptins, and empagliflozin were also suggested for this population [113]. The guidelines by the Research Society for the Study of Diabetes in India recommended diabetes treatment depending on hypertension severity, which includes ARB, ACEi, calcium channel blockers, beta blockers, and diuretics (thiazides). Angiotensin receptor blockers are safe and well-tolerated in reducing CV, cerebrovascular, and renal complications; ARBs are suggested over ACEis when BP control is primary in people with diabetes. Angiotensin receptor blocker or ACEi in combination with SGLT-2i has shown better BP control and eGFR in people with T2D [93]. Indian guidance (2018) on CV and renal morbidity management in T2D and KDIGO guideline (2024) recommended ARB or ACEi alone but not in combination for CKD treatment [13,38]. A more detailed guideline recommendation is provided in Table 1.

**Table 1:** Guideline recommendations for the management of diabetes with cardiovascular and/or renal complications Strong consensus recommendations (based on the level of evidences, as given by the respective guidelines) are highlighted in bold ACC/AHA, American College of Cardiology/American Heart Association; ACEi, angiotensin-converting enzyme inhibitor; ARB, angiotensin receptor blocker; ASCVD, atherosclerotic cardiovascular disease; BP, blood pressure; CAD, coronary artery disease; CCB, calcium channel blocker; CVD, cardiovascular disease; CV, cardiovascular; DM, diabetes mellitus; DPP-4i, dipeptidyl peptidase-4 inhibitors; eGFR, estimated glomerular filtration rate; ESC, European Society of Cardiology; ESKD, end-stage kidney disease; GLP-1RA, glucagon-like peptide-1 receptor agonist; HbA1c, hemoglobin A1c; HF, heart failure; HFpEF, heart failure with preserved ejection fraction; HFrEF, heart failure with reduced ejection fraction; hHF, hospitalization for HF; KDIGO, Kidney Disease: Improving Global Outcomes; LDL-C, low-density lipoprotein-cholesterol; LVEF, left ventricular ejection fraction; MRA, mineralocorticoid receptor antagonists; NYHA, New York Heart Association; PCSK9, proprotein convertase subtilisin-kexin type 9; RSSDI, Research Society for the Study of Diabetes in India; SGLT-2i, sodium-glucose cotransporter-2 inhibitor; T2D, type 2 diabetes; UACR, urinary albumin-to-creatinine ratio; MI, myocardial infarction; CKD, chronic kidney disease

Reference	Guideline	Recommendations for management of DM with CVD and/or kidney disease
[87,114]	American Diabetes Association Professional Practice Committee (2025)	SGLT-2i and GLP-1RA
		SGLT-2i and GLP-1 RA to reduce albuminuria
		SGLT-2i (if eGFR≥20 mL/minute/1.73m^2^) in people with T2D and CKD to reduce CVD risk
		SGLT-2i or GLP-1RA in people with T2D and established ASCVD or renal disease
		SGLT-2i in people with T2D and established HFpEF or reduced HFrEF
		SGLT-2i in people with T2D and established ASCVD, multiple ASCVD risk factors, or CKD
		Statin/non-statin therapy
		Initiate statin therapy in people with diabetes with ASCVD risk factors, aged 20-39 years, in addition to lifestyle therapy
		Moderate-intensity therapy in people with diabetes without ASCVD, aged 40-75 years, in addition to lifestyle therapy
		Moderate-intensity statin therapy in people with diabetes, aged >75 years
		High-intensity therapy in people aged 40-75 years with diabetes, LDL-C of ≥70 mg/dL (≥1.8 mmol/L), and ≥1 ASCVD risk factor
		Ezetimibe or PCSK9 inhibitor with maximally tolerated statin therapy in people with diabetes, LDL-C of ≥70 mg/dL (≥1.8 mmol/L), and ASCVD risk, aged 40-75 years
		Icosapent ethyl in addition to statin therapy in people with ASCVD or other CV risk factors and controlled LDL-C but elevated triglycerides (150-499 mg/dL), to reduce CV risk
		Other antidiabetic drugs
		Metformin in people with T2D and stable HF, if eGFR>30 mL/minute/1.73 m^2^
		ACEi or ARB
		First-line therapy for hypertension in people with diabetes and CAD or diabetes and UACR (≥300 mg/g creatinine)
		Combination of ACEi and ARB and combination of ACEi or ARB with direct renin inhibitors are not recommended
		MRA
		Non-steroidal MRA (if eGFR≥25 mL/minute/1.73m^2^) in people with T2D and CKD to reduce CVD risk
		Non-steroidal MRA in people with T2D and CKD treated with ACEi or ARB (maximum tolerated dose) to improve CV outcomes and reduce the risk of CKD progression
[105]	The ESC guideline (2023)	SGLT-2i and GLP-1RA
		SGLT-2i in people with T2D with multiple ASCVD risk factors or established ASCVD
		SGLT-2i in people with T2D and HFrEF (NYHA classes II-IV) to reduce the risk of hHF and CV death
		SGLT-2i in people with T2D and LVEF of >40%
		SGLT-2i in people with T2D and CKD with eGFR of ≥20 mL/minute/1.73 m^2^
		GLP-1RAs in people with T2D at risk of or with HF
		GLP-1RA in people with diabetes and CKD (eGFR of >15 mL/minute/1.73 m^2^) to achieve adequate glycemic control with low risk of hypoglycemia and beneficial effects on CV risk and albuminuria
		SGLT-2i or GLP-1RA in people with T2D and ASCVD, independent of baseline/target HbA1c and concomitant glucose-lowering medication
		SGLT-2i and GLP-1RA in people with T2D without ASCVD and 10-year CVD risk
		DPP-4i
		Sitagliptin and linagliptin in people with T2D at risk of or with HF
		Saxagliptin is not recommended in people with T2D and at risk of HF or with previous HF
		Statin/non-statin therapy
		PCSK9 inhibitor for people with diabetes and at very high CV risk, with persistently high LDL-C levels, despite having statin therapy (maximum tolerated dose) combined with ezetimibe, or having statin intolerance
		Statin/ezetimibe combination in people with diabetes and CKD
		Other antidiabetic drugs
		Metformin in people with T2D and ASCVD
		Pioglitazone in people with T2D and ASCVD, without HF
		Basal insulins (glargine and degludec) in people with T2D at risk of or with HF
		ACEi or ARB
		Sacubitril/valsartan or ACEi in people with T2D and HFrEF (NYHA classes II-IV) to reduce the risk of hHF and death; in case of sacubitril/valsartan or ACEi intolerance, ARBs are recommended
		Maximum tolerated dose of an ACEi or ARB in people with diabetes and CKD
		Combination of an ARB and an ACEi is not recommended
		MRA
		MRAs in people with T2D and HFrEF (NYHA classes II-IV) to reduce the risk of hHF and death
		Finerenone in addition to ACEi or ARB in people with T2D and eGFR of >60 mL/minute/1.73 m^2^ with UACR of ≥300 mg/g or eGFR of 25-60 mL/minute/1.73 m^2^ and UACR of ≥30 mg/g
[115]	The ACC/AHA guideline (2019)	SGLT-2i and GLP-1RA
		SGLT-2i or GLP-1RA in people with T2D and ASCVD risk factors
		Statin therapy
		Moderate-intensity statin therapy in adults (40-75 years) with DM, regardless of estimated 10-year ASCVD risk
		High-intensity statin therapy in adults with DM and multiple ASCVD risk factors
[38]	KDIGO guideline (2024)	SGLT-2i and GLP-1RA
		SGLT-2i in people with T2D, CKD, and eGFR of ≥20 mL/minute/1.73 m^2^
		Long-acting GLP-1RA in adults with T2D and CKD, who have not achieved individualized glycemic targets despite using metformin and SGLT-2i
		Statin therapy
		In adults (18-49 years) with CKD but not treated with chronic dialysis or kidney transplantation and with one or more of the following: MI or coronary revascularization, DM, or stroke
		Estimated 10-year incidence of coronary death or nonfatal MI of >10%
		ACEi or ARB
		In people with diabetes and CKD and moderate-to-severe increased albuminuria
		MRA
		In adults with T2D and eGFR of >25 mL/minute/1.73 m^2^, normal serum potassium concentration, and albuminuria (>30 mg/g)
[93]	RSSDI guideline (2022)	SGLT-2i
		SGLT-2i in combination with ACEi and ARB in people with T2D and CKD, to reduce the composite kidney outcome
		SGLT-2i in people with diabetes for reno-protection
		DPP-4i
		Recommended for people with diabetes for reno-protection
		ACEi or ARB
		First-line treatment agents for people with diabetes and hypertension
		ARB in combination with CCBs or diuretics in people with DM, to reduce the risk of CVDs and renal disorders
		ARB monotherapy or combination therapy with CCBs in people with DM to control BP
		Both ARB and ACEi in people with diabetes and hypertension, to reduce the risk of ESKD
		ACEi/ARB alone or in combination with SGLT-2i in people with T2D for controlling BP and eGFR
		Diuretics
		Thiazides alongside ACEi or ARBs in people with DM for the management of hypertension
		Thiazides and beta blockers are not recommended in people with diabetes and hypertension, as they cause CV events and hyperglycemia
		Thiazide in eGFR of ≥40 mL/minute/1.73 m^2^
		Loop diuretics in eGFR of ≤40-50 mL/minute/1.73 m^2^
[116]	Indian Society of Hypertension guideline (2023)	SGLT-2i and GLP-1RA
		SGLT-2i in people with T2D to reduce CVD/CKD risk and HF and in diabetic/nondiabetic nephropathies when eGFR is 20 or 25 mL/minute/1.73^2^
		SGLT-2i or GLP-1RA in obese people with diabetes and hypertension, to reduce body weight and BP
		Statin therapy
		Statins in people with hypertension and dyslipidemia to prevent CVDs
		ACEi or ARB
		ACEi and ARB in people with proteinuria or microalbuminuria
		ACEi or ARB (or beta blockers) in people with hypertension and CAD

## Conclusions

Modifiable risk factors, including diabetes, dyslipidemia, and hypertension, increase inflammatory markers and ADMA, decrease nitric oxide bioavailability, and result in endothelial dysfunction. This in turn causes hypertension or albuminuria, leading to atherosclerosis-mediated CV or kidney damage/failure. The timely diagnosis and management of the vascular complications at “prevent” and “regress” stages of the diabetes-kidney-heart continuum are crucial for delaying the irreparable consequences of the “retard” stage. Moreover, individual risk stratification must be prioritized during the selection of first-line therapy and transition to other suitable therapies to delay the continuum. Metformin and SGLT-2i are the first-line treatment for patients with T2D and CKD (if eGFR allows). In case the HbA1c goal is not achieved with the first-line therapies, GLP-1RA can be used in people with diabetes but without congestive HF.

Secondary prevention with SGLT-2i or GLP-1RA in people with T2D, CVD, and CKD (eGFR of ≥20 mL/minute/1.73 m^2^) can be considered for reducing both CV risk and albuminuria. Dipeptidyl peptidase-4 inhibitors provide reno-protection by potentially reducing UACR and a decline in eGFR. Monotherapy of ACEi or ARB or a combination therapy with SGLT-2i is recommended in people with T2D for controlling BP and eGFR. The use of RAS blockers reduces the risk of hHF or death in people with T2D and CVD (including HF) or CKD (including moderate-to-severely increased albuminuria). Similarly, MRAs reduce the risk of hHF and death in T2D patients having HF with reduced EF. Lipid lowering with statin therapy is recommended in people with diabetes and established CVD. In advanced CKD patients, the use of fibrate, omega-3 supplementation, bile acid sequestrants, and PCKS9 inhibitor has shown promising effects in reducing triglyceride, LDL-C, MACE, and CV death. In addition to the systematic approach, improving treatment adherence is equally important, with a focus on minimizing prescription costs, active monitoring, and providing health literacy to obtain greater treatment outcome.
